# The Oxford Face Matching Test: Short-form alternative

**DOI:** 10.1177/17470218231182933

**Published:** 2023-06-22

**Authors:** Mirta Stantić, Jacob Knyspel, Akhina Gaches, Yining Liu, Geoffrey Bird, Caroline Catmur

**Affiliations:** 1 Department of Experimental Psychology, University of Oxford, Oxford, UK; 2 Department of Psychology, Institute of Psychiatry, Psychology & Neuroscience, King’s College London, London, UK; 3 Social, Genetic and Developmental Psychiatry Centre, Institute of Psychiatry, Psychology & Neuroscience, King’s College London, London, UK

**Keywords:** Oxford Face Matching Test, face matching, face perception, reliability, short-form, validity

## Abstract

A recently published test of face perception, the Oxford Face Matching Test, asks participants to make two judgements: whether two faces are of the same individual and how perceptually similar the two faces are. In this study, we sought to determine to what extent the test can be shortened by removing the perceptual similarity judgements and whether this affects test performance. In Experiment 1, participants completed two versions of the test, with and without similarity judgements, in separate sessions in counterbalanced order. The version without similarity judgements took approximately 40% less time to complete. Performance on the matching judgements did not differ across versions and the correlation in accuracy across the two versions was comparable with the originally reported test–retest reliability value. Experiment 2 validated the version without similarity judgements against other measures, demonstrating moderate relationships with other face matching, memory, and self-report face perception measures. These data indicate that a test version without the similarity judgements can substantially reduce administration time without affecting test performance.

A novel face matching measure, the Oxford Face Matching Test (OFMT; Stantić et al., 2022a), was recently introduced as an alternative to existing face perception tests. It was specifically designed to be usable across typical and atypical populations (Stantić et al., 2022b) as well as sensitive to the full range of individual differences. The long version of the test includes 200 trials (100 matching and 100 mismatching). In each trial (see Figure 1a), participants are asked: (1) whether two faces presented simultaneously were of the same person or different people and (2) to explicitly estimate the perceptual similarity of the faces (ranging between 0 [*very dissimilar*] and 100 [*very similar*]). The first of these questions provides the OFMT accuracy score, while the second can be used to compare participant perceptual similarity judgements with those generated by computer vision algorithms. These explicit estimates of perceptual similarity can be used to dissociate face matching (the ability to determine whether two facial images depict the same, or different, facial identities) from face perception (the ability to form perceptual representations of faces). This dissociation has potential theoretical and practical utility, e.g., it has been used to show that individuals with developmental prosopagnosia exhibit impairments in face perception, face matching, and face memory (Stantić et al., 2022c), whereas individuals with autism have difficulties with face perception and face memory, but intact face matching (Stantić et al., in press), suggesting that different intervention strategies may be useful to ameliorate face processing difficulties in these two groups.

**Figure 1.**
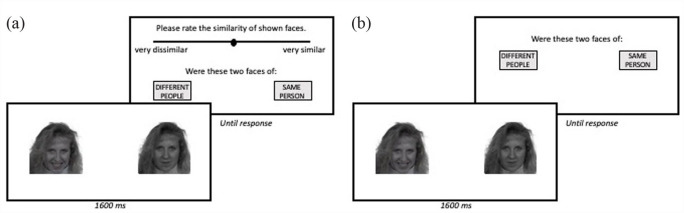
(a) Trial structure with similarity judgements. (b) Trial structure without similarity judgements.

However, compared with other face matching measures (Glasgow Face Matching Test, Burton et al., 2010; Pairs Matching Test, Bate et al., 2018; Kent Face Matching Test, Fysh & Bindemann, 2018), the OFMT is longer to administer, taking about 20–25 min (compared with, e.g., 3–4 min for the Glasgow Face Matching Test; Burton et al., 2010). This is in part due to a larger number of trials and in part due to the necessity for both responses to be provided. As face matching measures are often administered as part of a battery of face tests, this administration time may be problematic. A previous attempt to reduce administration time involved reducing the number of trials, but this resulted in relatively noisy estimates of performance (Stantić et al., 2022a). Providing researchers do not require the ability to distinguish between face perception and face matching, it might be possible to administer the OFMT without the perceptual similarity question in an attempt to reduce administration time. However, whether the OFMT can be administered without the question about perceptual similarity, or if its exclusion would fundamentally alter test performance, remains an empirical question. Here, therefore, we investigate the consistency of test performance when administered with and without similarity judgements (Experiment 1) and validate the resulting short-form OFMT against other face processing measures (Experiment 2).

## Experiment 1

### Method

A total of 45 participants (*M* ± *SD* age = 27 ± 9 years, 20 female, 24 male, 1 other) completed the 200-trial version of the OFMT online via gorilla.sc twice across two testing sessions 24–48 hr apart, such that one administration of the test required similarity judgements to be made, and the other did not (see Figure 1). The order of testing sessions was counterbalanced across participants.

During development of the OFMT, pairs of stimuli were selected from two databases of face images, one held by the authors and the Face Recognition Technology (FERET) data set (made publicly available by DARPA; Phillips et al., 1997, 1998). Images were kept in their original naturalistic state, with background removed and shown in greyscale, but without any further cropping of external features or processing applied. All images were of Caucasian people and without glasses (to avoid reliance on external features in making a matching decision). Images were of faces presented frontally without an explicit presentation of emotion (e.g., faces are either neutral or slightly smiling).

During test preparation, over 3 million face pairs were assessed for similarity by three different algorithms. The resulting similarity index placed each face pair into a difficulty bin (ranging from 1 to 20). Finally, five matching (“same”) and five mismatching (“different”) image pairs were selected from each bin at random for final presentation in the OFMT. This results in a total of 200 stimulus pairs (100 matching or “same” and 100 mismatching or “different”) in the test.

On each trial of the OFMT, images of naturalistic uncropped faces are shown side by side for 1600 ms, and participants are required to indicate whether the images are of the same person or different people, as well as, for the long-form version, indicating the similarity of the two faces. The 200 trials are split into four blocks, with trials within blocks presented at random. The task also includes 10 attention check trials, designed to be easy even for those with severe face processing impairments, distributed across the four blocks. Of these attention checks, five “same” pairs show exactly the same images of a face, while five “different” pairs show faces of different genders.

Participants were compensated with course credit or a small monetary incentive for participation and both experiments were approved by the local ethics committee. For both experiments, participants who failed to complete any task or who failed two or more of the OFMT attention check trials were excluded prior to data analysis.

### Results

**Figure 2.**
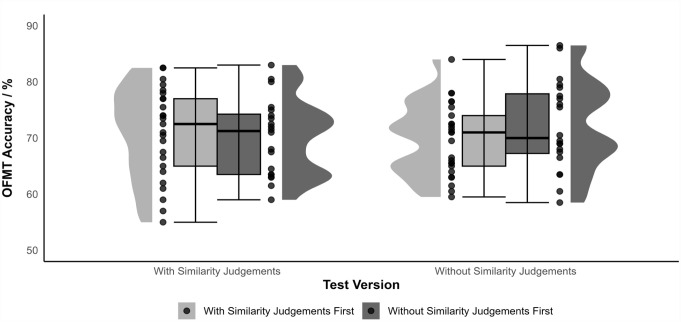
Performance on the two versions of the test (with and without similarity judgements) across the two version orders (version with similarity judgements first, version without similarity judgements first). Box plots indicate the first and third quartiles, with the median indicated by the bold line; whiskers indicate the minimum and maximum values.

No participants were excluded for Experiment 1. Performance on the two versions of the test was broadly similar (matching accuracy with similarity judgements: *M* ± *SD* = 70.6 ± 7.5%; without similarity judgements: 70.8 ± 7.2%). Accuracy data were submitted to a 2 (version: with, without similarity judgements) × 2 (version order: with first, without first) mixed analysis of variance (ANOVA). Neither the main effect of version nor the main effect of version order were significant, but an interaction was observed (*F*(1, 43) = 5.12, *p* = .029, $\eta_{p}^{2}=0.106$ ) consistent with a slight practice effect (see Figure 2; cf. Stantić et al., 2022a, Experiment 3). The simple effect of version was not significant for either version order.

The correlation between performance on the two versions of the test was strong, *r*(44) = .760, *p* < .001, and of a similar magnitude to previously reported test–retest reliability correlations for this test (Stantić et al., 2022a). Importantly, test duration was substantially shorter for the version without similarity judgements (*Mdn* = 14 min, Q1 = 11 min, Q3 = 21 min, interquartile range [IQR] = 10 min) compared with the version with similarity judgements (*Mdn* = 23 min, Q1 = 17 min, Q3 = 29 min, IQR = 12 min; *Z*_Wilcoxon_ = 3.74, *p* < .001).

Participants may use a variety of response strategies when completing a face matching task (Megreya, 2018; Towler et al., 2021), and it is possible that the use of such strategies in this task may be affected by the presence or absence of the similarity judgements. We therefore repeated the mixed ANOVA above with the addition of a further within-participant factor of trial type (matching [“same”], mismatching [“different”]). Accuracy on these two trial types corresponds to hits and correct rejections, respectively. Accuracy rates on the two trial types were similar across the two versions of the test: matching with similarity judgements: *M* ± *SD* = 69.8 ± 15.1%; without similarity judgements: 68.0 ± 14.3%; mismatching with similarity judgements: *M* ± *SD* = 71.3 ± 21.1%; without similarity judgements: 73.4 ± 18.9%. There was no main effect of trial type nor interactions with the factors of test version or version order.

## Experiment 2

Experiment 2 sought to validate the short-form version of the OFMT against other commonly used face processing measures, in a similar manner to the validation of the long-form OFMT (Stantić et al., 2022a).

### Method

A total of 151 participants started the testing session. Data from three participants were removed due to technical issues meaning images were not presented accurately on more than five trials of the OFMT and/or the Cambridge Face Memory Task (CFMT). One data set was removed due to incorrect responses on more than two attention check trials on the OFMT, and one due to the participant reporting a vision problem which was not corrected. The remaining 146 participants (*M* ± *SD* age = 19.8 ± 2.3 years, 134 female, 10 male, 2 other) completed the short-form version of the OFMT (without similarity judgements) and three other face processing measures, all in counterbalanced order, in one in-person testing session.

The CFMT (Duchaine & Nakayama, 2006) measures memory for unfamiliar faces. Participants learn six target faces at the beginning of the test, after which they are tested on three-alternative forced-choice trials. On each trial, two images are distractors and one is an image of a learned target identity. The test is divided into 3 stages of increasing difficulty, involving 18 test trials with no change of viewpoint or lighting, 30 trials with viewpoint and lighting changes, and 24 trials with viewpoint and lighting changes along with the addition of visual noise.

The Glasgow Face Matching Task (GFMT; Burton et al., 2010) assesses unfamiliar face matching ability. Participants are shown two faces of either the same individual (match trials) or different individuals (mismatch trials) for an unlimited amount of time and asked to determine if faces belong to the same person or different people. The short-form version used here consists of 20 match and 20 mismatch trials, presented in a random order.

Finally, the 20-Item Prosopagnosia Index (PI-20; Shah et al., 2015) is a self-report questionnaire used as a screening tool to identify people with difficulties in face recognition. It has previously been validated against the CFMT and has been shown to distinguish those with prosopagnosia from the neurotypical population. The survey consists of 20 items on which respondents can report face recognition difficulties in everyday life, with higher scores representing more severe face recognition difficulties.

### Results

Performance on the four measures is presented in Table 1. Mean performance scores were consistent with the originally reported means for the CFMT (*M* = 57.9, Duchaine & Nakayama, 2006), GFMT (*M* = 81.3, Burton et al., 2010), and PI-20 (*M* = 38.9, Shah et al., 2015).

**Table 1.** Performance on face processing measures in Experiment 2.

Measure	*M*	*SD*	Minimum	Maximum
OFMT Short Form	75.8	5.8	58.5	87.5
Matching	69.9	13.5	24.0	96.0
Mismatching	81.9	9.8	48.0	98.0
CFMT	52.9	9.4	30	70
GFMT	82.0	11.7	52.5	100.0
PI-20	40.1	9.7	21	71

Scores on OFMT and GFMT represent percentage accuracy.

OFMT: Oxford Face Matching Test; CFMT: Cambridge Face Matching Task; GFMT: Glasgow Face Matching Task; PI-20: 20-Item Prosopagnosia Index.

Simple correlations were performed between scores on the OFMT Short Form and each of the other face processing measures. As measures other than the OFMT were not normally distributed, Spearman’s ρ was used. Significant correlations were found between the OFMT Short Form and GFMT: ρ_144_ = 0.263, *p* = .001; CFMT: ρ_144_ = 0.463, *p* < .001; and PI-20: ρ_144_ = −0.305, *p* < .001. These correlations are broadly consistent with those found between the long-form OFMT and these measures in Studies 1–3 of Stantić et al. (2022a): in that paper, the correlation with the GFMT was *r* = .46; correlations with the CFMT ranged from *r* = .32 to *r* = .41; and with the PI-20 from *r* = −.14 to *r* = −.22.

The same analyses were repeated for percentage accuracy on matching and mismatching trials. Significant correlations were found between accuracy on matching trials on the OFMT Short Form and scores on the CFMT: ρ_144_ = 0.323, *p* < .001 and PI-20: ρ_144_ = −0.211, *p* *=* .010 and between accuracy on mismatching trials on the OFMT Short Form and scores on the GFMT: ρ_144_ = 0.390, *p* < .001. This pattern of relationships between performance on matching and mismatching trials for the OFMT, and GFMT performance, is consistent with that reported in Stantić et al. (2022a), suggesting that the removal of the similarity question does not have a substantial impact on the relationship between performance on the OFMT and on other face processing measures.

## General discussion

We sought to investigate whether the OFMT can be substantially shortened for ease of administration. Our results indicate that matching performance remains consistent regardless of whether the similarity question is asked. Eliminating the similarity question substantially reduces the length of the full test, and the administration of the 200 trials of the OFMT takes, on average, less than 15 min without this question.

The relationships between performance on the short-form OFMT and performance on other face processing measures were consistent with those reported for the long-form OFMT in the initial validation study (Stantić et al., 2022a), with small to moderate relationships observed with all measures. This is also consistent with the magnitude of relationships reported between other face processing tests (e.g., Verhallen et al., 2017). Performance on the short-form OFMT was numerically higher in Experiment 2 than Experiment 1; this likely reflects the in-person versus online nature of the testing, as a similar discrepancy was observed in Stantić et al. (2022a, Study 1 vs Study 2).

These findings have practical significance for all researchers interested in using the OFMT. They show that face matching performance can be measured using the OFMT without explicitly asking participants to estimate the perceptual similarity of presented faces. It is therefore expected that indicative OFMT scores across ages and genders (Stantić et al., 2021) can be used as guides for performance with or without similarity judgements. It should, however, be noted that we did not test neurodiverse populations (e.g., super-recognisers, developmental prosopagnosics, autistic participants) in this study, and it is possible that such populations might show different patterns of results to the present sample.

In conclusion, although some researchers (e.g., those interested in dissociating face perception and face matching) might find the deployment of the long-form OFMT with similarity judgements more suitable for their purposes, these results show that its use without similarity judgements significantly shortens the length of the test without affecting matching performance.
